# The Role of Lead Exposure on Attention-Deficit/ Hyperactivity Disorder ‎in Children: A Systematic Review

**Published:** 2016-01

**Authors:** Maryam Daneshparvar, Seyed-Ali Mostafavi, Maryam Zare Jeddi, Masud Yunesian, Alireza Mesdaghinia, Amir Hossein Mahvi, Shahin Akhondzadeh

**Affiliations:** 1 Department of Environmental Health Engineering, School of Public Health, Tehran University of Medical Sciences, Tehran, Iran; 2 Psychiatry and Psychology Research Center, Tehran University of Medical Sciences, Tehran, Iran; 3 Department of Environmental Engineering, Texas A&M University, Kingsville, Texas, USA; 4 Institute for Environmental Research (IER), Tehran University of Medical Sciences, Tehran, Iran; 5 Center for Air Pollution Research (CAPR), Institute for Environmental Research (IER), Tehran University of Medical Sciences, Tehran, Iran; 6 Psychiatric Research Center, Roozbeh Hospital, Tehran University of Medical Sciences, Tehran, Iran

**Keywords:** ADHD, Attention Deficit Disorder with Hyperactivity, Blood Lead Level, Lead Poisoning, Nervous System, Childhood

## Abstract

**Objective: **Attention-Deficit/Hyperactivity Disorder (ADHD) is one of the most common behavioral ‎disorders in children effecting the families and society. This systematic review examined ‎the literature on the role of lead exposure in children with ADHD‏ ‏symptoms. Articles were ‎analytically compared, focusing on the methodology used to assess exposure and‏ ‏adverse ‎effects‏ ‏on children with ADHD. ‎

**Method:** Using the search strategy from six databases (Pub Med, PsycINFO, Web of Science, SID, ‎IRAN Medex, IRAN DOC), hand searching in key journals, list of references of selected ‎articles and gray literature, without time and language limitation, articles up to May 2014 ‎were entered into this review. In this review, 1,387 articles were acquired at the primary ‎search. Study selection and quality assessment processes were done based on Cochrane ‎library guidelines. After assessing the quality and inclusion and exclusion criteria, 18 articles ‎were selected and entered into the data synthesis.‎

**Results:** Blood Lead level (BLL) of less than 10µg/dL in children has been attributed to at least one ‎type of ADHD i.e., Combined / Inattentive / Hyperactive-Impulsive. The results of this ‎study revealed that in 16 out of the 18 studies, a significant association was found between ‎BLL and one of the types of ADHD.‎

**Conclusion**: Based on the findings of this study, even the BLL of less than the action level of 10µg/dL, ‎chosen by Centers for Disease Control and Prevention (CDC), may affect children with ‎ADHD.‎

Attention deficit / hyperactivity disorder (ADHD) is one of the most commonly diagnosed ‎psychiatric disorders in children. In addition, it is probably the most common chronic ‎condition undiagnosed in adults (1, 2). According to the American Psychiatric ‎Association’s Diagnostic and Statistical Manual Fifth Edition, 3 to 7% of children in school ‎and 2 to 4% of the adult population have ADHD (3, 4). The global statistics show that ‎about 10.1% of the world’s population has ADHD (5), making this disorder an important ‎health issue. This disorder continues to about 50 to 80% of teenagers and 15 to 65% of ‎adults (6, 7). Moreover, ADHD is more prevalent in boys than girls (9). Children are more ‎exposed to psychiatric disorders such as antisocial personality (9), depression, unipolar ‎depression (10), bipolar (11), anxiety, autism, learning disabilities, emotional disturbance, ‎and fiery temperament (9, 12 and 13). The tendency for drug use and addiction increases in ‎adults if the disorder is not diagnosed and treated at a younger age (14). Some ADHD ‎symptoms disappear with time, but the symptoms such as lack of concentration are constant ‎and a person will be show them throughout their lifetime (13). Therefore, considering the ‎implications, the efforts for early diagnosis of the disease is crucial, and identifying the ‎contributing factors is of prime importance to prevent ADHD.‎

Biological and environmental factors are the pathogenesis of this disorder, including head ‎injury, a decrease in the prefrontal cortex, and toxins and chemicals found in the ‎environment (15). Although evidence shows that ADHD is a familial problem, many ‎environmental risk factors such as exposure to heavy metals, Dietary factors, environmental exposure to ‎dangerous chemicals such as bisphenol A, polycyclic aromatic compounds, pesticides ‎intensify or accelerate the progression of this disease (16-18).‎

Lead (Pb) metal as a neurotoxin has been causing abnormal behavior in children, and many ‎studies have examined the relationship between exposure to heavy metals and other harmful ‎environmental factors in the pathogenesis (19-21). Since 1960, the CDC recommended‏ ‏levels for blood lead levels in children have been steadily reduced, and this has been due to ‎increased researches showing that it has negative effects on health. Currently, the children's ‎blood lead level (BLL) is set to 10 micrograms of lead per deciliter of blood (µg/dL), and it ‎has not been changed since 1991 (22). The CDC action level set for lead may be outdated ‎now as recent research indicates that adverse health effects may be associated with blood ‎lead levels below 10µg/dL. Many studies have been conducted on cognitive problems in ‎children including reduced IQ scores, math, reading, verbal memory, and spatial ability, ‎with blood lead levels below 10µg/dL. Recent trends showed a 3% increase in the ‎diagnosis of attention deficit/ hyperactivity disorder (ADHD) in each year from 1997 to ‎‎2006 (23). Researchers are investigating whether exposure to lead contributes to an increase ‎in the number of ADHD cases. ‎

A systematic review was conducted to have a clear answer and deep understanding of the ‎topic of concern. Among all the heavy metals, Lead is one of the significant heavy metals ‎causing mental illness. In order to have a clear and deep understanding of the effects of ‎lead on the mental health of children, lead was selected as the main element for the ‎systematic review in this paper. The aim of this study was to systematically review all the ‎studies showing the relationship between ADHD symptoms and blood lead levels below ‎‎10µg/dL in children. The findings of these studies also account for the gaps and research ‎needs. Synthesizing the evidence related to ADHD symptoms in children with BLLs less ‎than 10µg/dL will help determine whether the current BLL is still appropriate for children ‎or not.‎

## Materials and Method

***Search Strategy*** ***:***

National and international databases, such as PubMed, PsycINFO, Web of Science, SID, ‎IRAN Medex, IRAN DOC, were searched. In addition, hand searching in key journals, list ‎of references of the selected articles in both English and Persian, and gray literature were ‎investigated. Moreover, Studies working on the age group of younger than 18 were selected ‎to be included into the systematic review. As per the CDC and government agencies ‎around the world, individuals who are younger than 18 years of age are considered as ‎children. The databases were thoroughly searched for articles with no time limit, until May ‎‎2014. Language limitation was not set as inclusion criteria. Animal studies were not ‎included in our review. In order to ensure that no relevant papers are lost, the lists of review ‎articles were also fully investigated.‎

***Keywords*** ***:***

The key words used for the search are divided into three categories as follows:"Attention ‎deficit disorder*";"Attention Deficit Disorder with Hyperactivity*‎‏"‏‎; Hyperactivity ‎Disorder*;‎‏"‏Attention Deficit Hyperactivity Disorder‏"*‏‎; Attention-deficit*; Attention ‎Deficit Disorder with Hyperactivity Risk Factor*; Inattention*; ADHD

The key words on lead metal were as follows: ‎

‎“Hazardous material*”, “Hazardous exposure*”, “Toxic Metal Exposure*”‎

‎“Toxic material*”, “Chemical hazard*”, Lead*, “Environmental toxicant*”‎

Key words on children: Infant*, Child*, Childhood*‎

The search terms with similar meanings were combined using the OR logic, and the search ‎terms were coupled using the AND logic. At the end, the search terms used for searching ‎the databases are as follows: ‎

‎(ADHD OR Hyperactivity disorder* OR Attention Deficit Disorder with Hyperactivity ‎Risk Factor* OR Attention Deficit Hyperactivity Disorder* OR Attention-deficit* OR ‎Attention Deficit Disorder with Hyperactivity* OR Attention deficit disorder* OR ‎Inattention*) AND (Lead* OR Hazardous material* OR Hazardous exposure* OR Toxic ‎Metal Exposure* OR Toxic material* OR Chemical hazard* OR Environmental toxicant*) ‎AND (Infant* OR Child* OR Childhood*) ‎

The search strategy was modified and customized for every database.‎

***Criteria for Inclusion and Exclusion*** ***:*** ***‎***

An article was included in our systematic review if it was ‎

1. An original article ‎
2. An article with the main subject of lead exposure in children (birth to 18 years)‎
3. An article studied on one of the three types of ADHD in children
4. (Inattentive, ‎Hyperactivity/Impulsivity, Combined)‎

At first, we evaluated the titles and abstracts of the retrieved articles to determine the initial ‎eligibility; and if necessary, the full papers were studied in detail in order to be selected for ‎the review.‎

**Figure 1:**
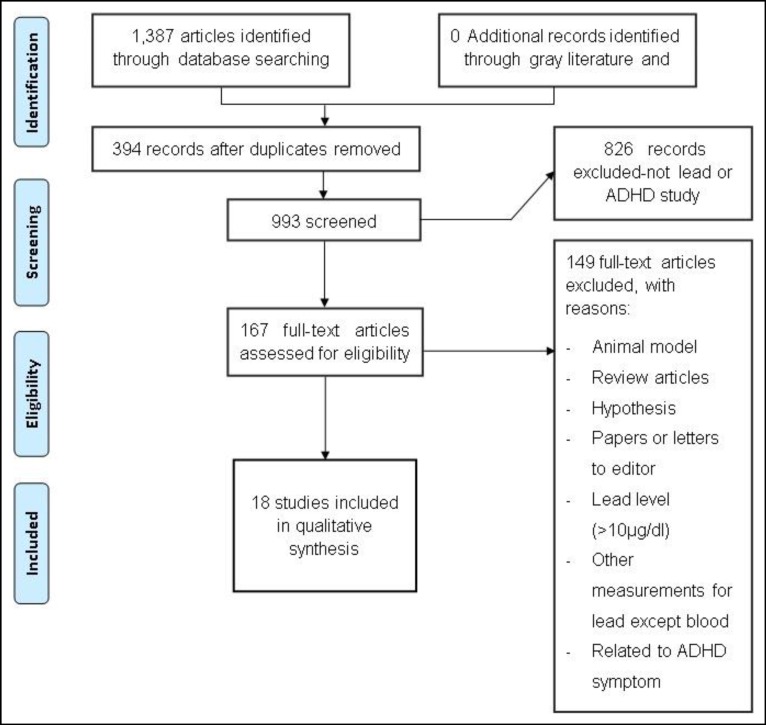
PRISMA 2009 Flowchart for the Included Studies of Lead Exposure in Children with ADHD (24)

To check the eligibility, those studies conducted on children under the age of 18, and those ‎on the relationship between blood lead levels and symptoms of ADHD (inattention, ‎hyperactivity and impulsivity) or types of ADHD with measured BLL<10µg/dL were ‎studied. Articles in which lead exposure was examined using urine, hair, nails and teeth ‎were excluded. Two researchers separately conducted the initial check on the titles and ‎abstracts of the papers and excluded the articles without the specific criteria. After a ‎detailed study, the remaining articles were included. Figure 1 demonstrates the search ‎strategy based on the PRISMA Flow Diagram.‎

***Assessment of the Quality of Articles*** ***:***

The quality assessment of the included articles was also a necessary task. There are many ‎international standards for quality measurement of articles like CASP, OTTAWA, ‎NEWCASEL, JADAD, CONSORT and STROBE (25). We used the STROBE ‎‎(Strengthening the Reporting of Observational Studies in Epidemiology) which included ‎quality standards for cross-sectional, case-control and cohort types of studies (26, 27).‎

The Checklist for STORBE is a list of 22 items; of which, eight items are related to quality ‎assessment and selected to be used in our study. Each of the quality assessment questions ‎was equivalent to 1 point, and the articles were divided into the following groups ‎depending on the points they obtained as per our Quality assessment Scale: ‎

‎

0-2 Points: Low quality ‎

‎3-5 Points: Medium quality‎

‎6-8 Points: High quality ‎

***Methods of Data Extraction*** ***:***

After screening databases and available resources, the initial articles were selected and their ‎data were extracted uniformly. The data extract form consisted of the author's name, year ‎of publication, place of study, the number of subjects examined, type of study, the amount ‎measured and the method of measuring BLLs and blood lead sample in the subjects, and ‎the technique used to determine ADHD. For establishing common grounds in all the ‎studies, the units of the measured BLLs were converted to microgram per deciliter (µg/dL).

**Table 1:** General Features and the Results of the Studies of Lead Exposure in Children with ADHD

**Author,** **Year,** **County**	**Study Type**	**Sample Description**	**Blood Lead Measurement Method**	**Blood Lead Levels of Sample**	**ADHD Symptom Measured**	**ADHD Symptom Measurement Method**
Canfield, 2003, U.S.	Cohort	170 infants recruited from a list of live births in urban hospitals in Rochester, NY.	Mean of 2 venous blood draws taken. Mean BLL at 48 months was used for analysis in study.	Mean BLL of all blood draws were <10 μg/dL. Average BLL at 48 months was 6.49 µg/dl; 48 months.	Attention	shape school task test at age 48 months (160/170 children)
Canfield 2004, U.S.	Cohort	Initial cohort was performed on 276 children. 174 remained at final	Venous blood tested Detection limit of BLL was 1 μg/dL.	Lifetime average BLL was 7.2μg/dL.	Attention	Children at age 5.5 years were tested with Shif Task Test.
Chiodo, 2004, U.S.	Cross- Sectional	337 African American children at 7.5 years of age, lead was unavailable for 91 children, and 9 BLL excluded from analysis due to very heavy prenatal alcohol exposure. Of the 237 children remained in the sample	Venous blood drawn at age 7.5 years by one of three phlebotomists at a children's hospital. Internal and external quality control measures taken. Limit of detection was 2 μg/dL.	Mean BLL was 5.4 ± 3.3 μg/dL. 92.4% BLL were below 10 μg/dL.	Inattention, Hyperactivity Impulsivity	Child's teacher completed the Barkely- DuPaul ADHD Scale and the Achenbach Child Behavior Checklist Teacher Report Form (TRF).
Braun, 2006, Mexican Americans, and non-Hispanic blacks	Cohort	Among children 4–15 years of age, 5,171 were available for analysis. Of the 4,704 eligible children 4–15 years of age, 344 (8.2%) had only parent reported ADHD and 154 (4.3%) reported stimulant medication use	Venous blood collected at beginning of study. No internal/external quality control measures were stated in the paper.	The limit of detection was reported to be 0.3 μg/dL (48) children had blood lead levels below this threshold. No detectable values were given values of 0.2 (0.3 divided by √2).	DSM-IV symptoms of ADHD–	Parent reported ADHD was based on the parent or guardian’s response.
**Author** **,Year,** **County**	**Study type**	**Sample description**	**Blood lead measurement method**	**Blood Lead Levels of sample**	**ADHD Symptom Measured**	**ADHD Symptom Measurement Method**
Min, 2007, South Korea	Cross- Sectional	Participants recruited at family health examination in an office in Seoul, Korea. 61 children ages 7 16 years (33 boys and 28 girls).	Venous blood drawn at time of study. External quality controls for blood lead were taken.	Mean BLL Was 2.94μg/dL; All BLL Below 5μg/dL.	Attention	Children completed the SPES-K (Korean version of SPES)
Chiodo, 2007, U.S.	Cross- Sectional	Laboratory testing was obtained from506 African American children born in the Detroit area between Sept. 1, 1989- Aug.	Venous blood drawn at age years at a children's hospital in Michigan. Internal and external quality control measures taken.	91.1% had BLL below 10μg/dL; Mean BLL was 5 ± 3 μg/dL.	Attention, Hyperactivity Impulsivity	Child completed Conner’s Continuous Performance Test (CPT) to measure impulsivity and inattention
Nigg, 2008, U.S	Case- Control	Final sample size: 150 children age 8-17 years: 53 non-ADHD controls, 47 ADHD inattentive type; 50 ADHD combined type.	Venous blood drawn at time of study. Analysis of BLL was done twice to ensure accuracy. Limit of BLL detection was 0.3μg/dL.	Maximum BLL was 3.4 μg/dL. Mean BLL for ages 8-11 Was 1.0 μg/dL.Mean BLL for ages 12-17 Was 1.03μg/dL.	Attention, Hyperactivity, ADHD diagnosis	Conners' Rating Scale Revised And ADHD Rating Scale. Completed by parents and Teachers. Parents also completed The K-SADS-E During interview With a clinician.
Wang, 2008, China	Case-Control	Chinese Children aged 4-12 were recruited There were 630 controls who were not diagnosed with ADHD and 630 were diagnosed with ADHD using DSM-IV-R Criteria.	Venous blood Samples collected. Limit of detection was 1.0 μg/dL. Each sample was analyzed for times to internal quality control.	Mean BLL for ADHD group was 8.77 μg/dL ± 3.89) and for controls was 5.76 μg/dL ± 3.39.	Diagnosed ADHD vs. Non-ADHD diagnosed controls	Children diagnosed after meeting criteria in the K-SADS-E modified to include the Aberrant Behavior Checklist completed by parents/ teachers.
**Author** **,Year,** **County**	**Study Type**	**Sample Description**	**Blood Lead Measurement Method**	**Blood Lead Levels of the Sample**	**ADHD Symptom Measured**	**ADHD Symptom Measurement Method**
Chandra mouli, 2009, U.K.	Cohort	All births from April 1, 1991- December 31, 1992 at Avon Health Authority in U.K. were eligible, resulting in total cohort of 14,062. 1135 participants from cohort were randomly selected to participate in study. Blood sample collected from488 participants.	Venous blood drawn at age 30 months. One sample differed by >2.5 μg/dL)	94% of sample had BLL below 10 μg/dL. He mean±SD BLL was 3.67±1.47	Hyperactivity	Parent and teacher of child completed the Strengths and Difficulties Questionnaire when child was 7 years old to measure Hyperactivity.
Froehlich, 2009, U.S	Cross-Sectional	National Health and Nutrition Examination Survey data collected from 3907 children age 8-15 years living in the US. Current BLL available for 2588 Children.	Venous blood drawn at time of survey completion. Limit of detection was 0.3μg/dL. No internal/external quality control measures were stated in the paper.	BLL range was 0.2 5 μg/dL. Children grouped into tertiles: first tertile (0.2-0.8 μg/dL), second tertile (0.9-1.3 μg/dL) and Third tertile (>1.3μg/dL).	DSM-IV criteria for ADHD	Child's caregiver completed The National Institute of Mental Health Diagnostic Interview Schedule for Children (DISC) to measure ADHD criteria.
Ha, 2009, South Korea	Cohort	2211 first and second grade students from 10 elementary schools from 6 South Korean cities were recruited. 1778 children ages 6-10 years had complete data (928 boys and 850 girls).	Venous blood collected at beginning of study. No internal/external quality control measures were stated in the paper.	Mean BLL was 1.8 μg/dL (in 2008 was 1.64 μg/dL and in 2010 was 1.60 μg/dL .	ADHD scores overall	Parents completed the Conner's Scale for ADHD symptom screening (higher score means higher severity of symptoms).
Cho, 2010, Korean cities	cross-sectional	A total of 667 children (age range 8–11) were recruited from nine schools in five Korean cities	Venous blood collected at beginning of study.	The mean blood lead level was 1.9 μg/dl ± .67 (range .53–6.16)	inattentive, hyperactive, and total scores	Teachers & parents completed the Korean version of the ADHD Rating Scales (KARS(
**Author, Year, County**	**Study type**	**Sample description**	**Blood lead measurement method**	**Blood Lead Levels of sample**	**ADHD Symptom Measured**	**ADHD Symptom Measurement Method**
Nicolescu, 2010, two Romanian cities were studied, namely Bucharest and Pantelimon	Cross-Sectional	83 children ages 8-12 years 49% girls and 51% boys. All children were asymptomatic for ADHD symptoms when recruited	Venous blood collected at beginning of study. Detection limit of BLL was 0.1μg/dL	Mean BLL in Bucharest was 3.2μg/dL; Mean BLL in Pantelimon was 5.1μg/dL	Inattention, Hyperactivity Impulsivity	Computerized German test called KITAP
Kim, 2010, Korean	Cross-Sectional	measured blood lead levels collected in 256 Korean children.	The detection limit for lead using method was 0.058 μg/dL.	Total mean blood lead level (N=205) with Mean±S.D= 5.40±8.4.	Inattentive hyperactive symptoms total score	Teachers ADHD Rating Scale (T-ARS)-IV.
Nigg, 2010, U.S	Case-Control	140 blood sample in children age 6-17 years	Venous blood collected at time Of study. BLL detection limit Was 0.3μg/dL.	Mean BLL Was 0.73 (SE=0.3) μg/dL. BLL range was <0.3 μg/dL to 2.2 μg/dL.	Inattention, Hyperactivity /Impulsivity, Diagnosed ADHD subtypes	K-SADS-E interview; Parents and teachers completed ADHD Rating Scale and Conners'-Revised.
Darugar, 2010, Iran	Case-Control	They randomize examine 100 children for case and 100 for control = 200 children	Venous blood was collected from each child( Blood was collected from the cubital vein of each child)	In Cases minimum BLL was 2.9 μg/dL and maximum was 18.6 μg/dL. Average BLL level for for cases was 7.2 μg/dL	Inattention Hyperactivity	Recognized by a psychologist based by Conner’s method
Boucher, 2012, Montreal	Cohort	Most of the participants (n = 208) were initially recruited under the auspices of the Cord Blood Monitoring Program 279(294 children and their mothers).	Venous blood collected at beginning of study. No internal/external quality control measures were stated in the paper.	Mean BLL Was Cord Pb (μg/dL): 4.7 and Current Pb (μg/dL): 2.7	A teacher report of at least six DSM-IV symptoms of ADHD -Inattentive type, Hyperactive impulsive type, ODD and CD.	ADHD (hyperactive impulsive type) based on the DBD (DSM-IV). The Teacher Report Form (TRF)
**Kim, 2013,** **USA**	**Case-Control**	**71 ADHD cases and 58 non-ADHD controls=129**	**Venous blood** **collected at time** **of study.**	**Mean BLL was In cases Geometric mean(range) is 1.29 μg/dL (0.28–9.99) and in controls 1.33 μg/dL (0.42–6.05) and blood Pb levels(< 5 mg/dL)**	**DSM-IV criteria**	**Child's teacher completed the Barkely-DuPaul ADHD Scale and the Achenbach Child Behavior Checklist Teacher Report Form (TRF).**

**Table 2:** Outcome and quality assessment of studies, limitations and benefits of Articles

**Author** **,Year,** **County**	**Strengths and Limitations of Study**	**Quality**
Chiodo, 2004, U.S.	**Strengths: **Controlled for various factors (maternal alcohol/drug use; socioeconomic status; maternal age, marital status, education, household; child sex), quality control measures taken for BLL measurements. **Limitations: **Only one-time blood lead measured which may not be true indicator of lifetime lead exposure; sample population only included children of African American mothers.	**High**
Braun, 2006, Mexican Americans, and non-Hispanic blacks	**Strengths:** Large sample size, large number of confounders determined (gender, child IQ, maternal education, home environment, maternal smoking, income); recent studies indicate that concurrent blood lead level is a stronger predictor of lead-associated IQ decrements than blood lead measured during early childhood, although some might argue that concurrent blood lead tests are not an adequate biomarker of a child’s lifetime exposure **Limitations: **the cross-sectional nature of the data makes it difficult to infer a causal relationship from observed associations; sample population only included children of Mexican Americans, and non-Hispanic blacks.	**High**
Min, 2007, South Korea	**Strengths: **External quality control measured for blood draws. **Limitations: **Confounding variable not included (SES, nutrition, environment of household). Study population recruited from a common district office which included all white collared workers. Only 1 blood measurement taken which may not be an indication of lifetime blood lead levels. Very small sample size.	**High**
Chiodo, 2007, U.S.	**Strengths: **Many control variables taken into account (prenatal alcohol and drug use, socioeconomic status, home environment, maternal IQ); internal and external quality control measures taken for BLL measurements; large sample size. **Limitations: **Only one-time blood lead measured which may not be true indicator of lifetime lead exposure; only African-American children were in the sample population and is not representative of population in the U.S.	**High**
Nigg, 2008, U.S	**Strengths: **Blood draws were analyzed twice to ensure accuracy, clinical diagnosis of ADHD types were known. **Limitations: **BLL measured only 1 time which may not be a good indicator of lifetime lead exposure; Hyperactive children may ingest more lead and therefore have higher BLL; not a random population sample.	**High**
**Author** **,Year,** **County**	**Strengths and Limitations of Study**	**Quality**
Chandra mouli, 2009, U.K.	**Strengths: **Large sample size, large number of confounders determined (gender, child IQ, maternal education, home environment, maternal smoking, income); quality control methods for BLL measurements were validated; **Limitations: **Selection bias due to higher income and higher education level of participants families; Only had lead data from 30 months which may or may not be representative of the child's BLL during critical age of development.	**High**
Wang, 2008, China	**Strengths: **Large sample size. Used ADHD diagnosed children and compared to non- ADHD children to compare BLL. Many covariates and confounders identified such as sex, low SES, age, family history of ADHD, maternal drinking, parent education level. Internal quality control measured taken for BLL measurement method. **Limitations: **Only one BLL measurement taken which may not be an accurate indication of lifetime BLL.	**High**
Froehlich, 2009, U.S	**Strengths:** Large sample size, large number of confounders determined gender, household income, age, race/ethnicity, postnatal tobacco exposure); used DSM-IV defined ADHD criteria. **Limitations**: Only 1 BLL was taken which may not reflect lifetime BLL exposure. No quality control measures for BLL measurements were stated.	**Medium**
Ha, 2009, South Korea	**Strengths: **Large sample population used. Large numbers of confounding factors were identified (education level of parents, household income, maternal smoking and alcohol consumption during pregnancy). **Limitations: **Only one BLL measured which may not be an accurate measurement of lifetime BLL. No internal/external quality control measures were stated.	**High**
Cho, 2010, Korean cities	**Strengths: **adjusted for age of the child, gender, paternal education level, maternal IQ, child’s IQ, and birth weight of the child **Limitations: **First, the cross-sectional nature of our data limits our ability to assess direct causal associations between environmental lead and tobacco smoke exposure and inattentive and hyperactive symptoms and neurocognitive performance. Second, a single measure of blood lead level may make it difficult to disentangle the effects of earlier childhood lead levels from current levels.	**High**
**Author** **,Year,** **County**	**Strengths and Limitations of Study**	**Quality**
Kim, 2010,	**Strengths: **they checked SES, parental education, ETS, smoking during pregnancy, and child gender **Limitations: **this study included the fact that the results may be confounded by unmeasured early neurodevelopmental patterns and the family history of ADHD in these children and limited information about these children's gestations and the home environment	**High**
Nicolescu, 2010,	**Strengths: **Various methods of measuring ADHD symptoms were used. **Limitations: **Possible confounders not controlled (home environment, maternal IQ), only 1 BLL measurement obtained which may not be a good indicator of lifetime BLL in child.	**High**
Nigg, 2010, U.S	**Strengths:** Clinical diagnoses of ADHD types were known; measured ADHD symptoms via multiple methods. **Limitations:** Only 1 blood lead measurement used which may not represent lifetime BLL;Not random population samples (sampling bias); hyperactive behavior may cause children to ingest more lead.	**High**
Darugar, 2010, Iran	**Strengths:** Differences in parents education and outcome of them are cofounding **Limitations:** Uncertainty of the reliability of the results, Only one BLL measured which may not be an accurate measurement of lifetime BLL.	**Low**
Boucher, 2012, Montreal	**Strengths:** The strengths of this study include ability to control for confounding by other contaminants present in seafood—specifically for confounding of the association between child Pb and outcomes by cord Hg. **Limitations:** this study is that the maternal report of substance use during pregnancy was obtained in many cases about a decade after delivery. Another limitation is that do not have formal diagnoses for ADHD.	**High**
Kim, 2013, USA	**Strengths:** relationship between lead (Pb) exposure and medically diagnosed attention deficit hyperactivity disorder (ADHD) in children. The role of mercury (Hg) and cadmium (Cd) exposures in ADHD development is even less clear. **Limitations:** small sample size, had difficulty matching ADHD cases with non-ADHD controls. Sapling was for African American and cucasian.	**High**

**Table 3:** The relationship between blood lead concentration and types of ADHD in the literature (Literature Review)

**ADHD or ADHD-related symptoms**	**pb**
**ADHD**	+ Braun 2006 +Wang 2008 + Froehlich 2009 + Nigg 2008, 2010 + Ha 2009 +Kim2013 +Boucher2012
**Attention problems**	+ chiodo 2007 +Cho 2010 +Kim 2010 +Min2007 + Chiodo2004 + Nigg 2008 +Canfield2003 -Canfield2004 -Boucher2012
**Impulsivity**	+ Nigg 2008 +Nicolescu 2010 - Chiodo 2007 -Chiodo2004 +Boucher2012
**Hyperactivity**	+ Chiodo 2007 +Nigg 2008 +Cho 2010 +Nicolescu 2010 +Boucher2012 -Chandramouli2009

" - " No significant association between lead exposure and types of ADHD

" + " A significant association between lead exposure and types of ADHD

## Results

After eliminating the duplicate articles and reviewing the titles and abstracts, 993 articles ‎were obtained for this review. After removing 826 unrelated records, 167 full texts were ‎assessed for eligibility (Figure 1). After reading the full text of the articles, according to the ‎inclusion and exclusion criteria mentioned in the methodology, 18 articles were included ‎into the systematic review.‎

The selected articles (n = 18) consisted of five case-control studies, six cohort studies and ‎seven cross-sectional studies. The number of subjects examined in these articles ranged ‎from a minimum of 61 to a maximum of 4,704 subjects. Overall, in the 18 articles, 12,195 ‎subjects were examined that included both male and female subjects. The youngest subject ‎was 30 months old and the oldest was 17years old. ‎

The methods of diagnosis (ADHD, hyperactivity, inattention and impulsivity) used were ‎different in the articles. The Conner’s Continuous Performance Test (CPT) was used in five ‎articles, KITAP in two articles, a new Korean Version of SPECS in three articles, and ‎DSM-IV in four articles. Three of the studies used questionnaires from the parents and ‎teachers report forms, and in the remaining two, a fusion of the above techniques was used ‎for diagnosis.‎

The results of the STROBE checklist with strengths and weaknesses of the examined ‎articles are reported in Table 2. From Table 2, 16 of the articles were of high quality. The ‎article by Froehlich (USA, 2009) had a medium quality and the article by Darugar (Iran, ‎‎2010) was of poor quality. The common limitation in all the articles (except for the ‎Canfield, 2003) was that the BLLs were measured only once. The range of the ‎concentration of lead in the blood for this study was between 0.2 and 8.77µg/dL (Table 1). ‎Table 3 shows the relation between exposure to lead and the type of ADHD associated ‎with each of the studied articles. A positive sign showed a significant relation whereas a ‎negative sign revealed an insignificant relation between exposure to lead and type of ‎ADHD.‎

## Discussion

Due to the potential negative consequences of ADHD and increased prevalence in the United States and other countries, for ADHD diagnosis, it is crucial to determine all the toxic environmental elements that cause this disorder. Lead, as a neurotoxin, can cause the formation of abnormal behavior by interfering with neurotransmitter (28). Experiments conducted on animals for lead exposure show a permanent alteration in glucocorticoid deregulation, hypothalamic-pituitary axis, and changed GABA-ergic (γ aminobutyric acid–containing) and dopaminergic systems, which is related to increase in anxiety and decrease in socializing behavior (29). Strange behaviors can occur under the influence of lead exposure and poisoning like anxiety, lack of inhibition of mental and social functioning, reduced IQ and learning problems causing seizures, coma and even death in some cases (30, 31).

In this systematic review, five of the articles reported the mean blood lead concentrations from 5-10µg/dL; of which, four articles showed a significant relationship between lead exposure and at least one type of ADHD. CDC reduced the standard BLL from 10µg/dL to 5µg/dL from 2012 to 2016. However, in 12 of the selected articles, even BLLs below 5µg/dL level were reported to have a significant positive relationship with at least one type of ADHD. In the study by Wang et al. (2008) (32), minimum blood lead levels, which can contribute to ADHD, was 5.765µg/dL. As indicated by Nigg et al. (33) and Braun et al. (34), the minimum BLL falls down to 1.26µg/dL and 2µg/dL. In one of the articles, the BLL values ranged from 2.5-5µg/dL, 5-10µg/dL to greater than 10µg/dL. The results of this study revealed that 94% of the 653 children who participated in the study had BLL of less than10µg/dL. The relationship between inattention and blood lead concentration was underlined in nine studies; of which, seven had a positive and significant correlation, and in the remaining two, the relation was insignificant. Nigg et al. (2008) and Braun and colleagues (2006) established that children with lead poisoning are more likely to develop behavioral inattention, and anxiety is also a common affect (28).

In six of the studies, the relationship between blood lead levels and hyperactivity disorder was investigated (Chandramouli et al., 2009, Chiodo et al., 2004, Chiodo et al., 2007, Nicolescu et al. , 2010, Nigg et al., 2008, Nigg et al., 2010); of which, five reported a positive association. Five articles focused on impulsivity disorder; of which, three showed positive effects. (Chiodo et al., 2004, Chiodo et al., 2007, Nicolescu et al., 2010, Nigg et al., 2010).

In some cases, confounding factors such as family economic status, parent’s education level, smoking habits, alcohol use, and other factors associated with blood lead levels were also considered. Some statistical models were used to determine the relationship between the concentration of lead and ADHD. A positive and statistically significant relationship was observed in both unadjusted and adjusted models. There were some exceptions due to the small sample size of the studies.

## Limitations

1. Most of the articles in the systematic review underlined the symptoms of ‎ADHD, but they did not concentrate on the diagnosis of ADHD, meaning ‎that having the symptoms may not necessarily disclose the actual ADHD ‎case.‎
2. In addition, the tests used to determine the type of ADHD are not ‎uniformed and standardized in all studies, and this may lead to differences ‎in data interpretation.

## Conclusion

Exposure to lead has negative consequences, which can be reduced by addressing the gaps ‎in the knowledge. The systematic review reveals that lead levels of less than 10µg/dL and ‎even less than 5µg/dL have significant effects on ADHD, Hyperactivity-Impulsive and ‎Inattentive disorder. To ensure the health and safety of children, the present permissive ‎blood lead levels need to be revised in the context of the recent evidence.‎

## Suggestions

1. In order to better clarity this systematic review, we need a meta-analysis. ‎
2. ‎Children exposed to lead in early stages of childhood are more prone to ‎problems in educational attainment even when the blood lead levels are less ‎than 5µg/dL. It would be favorable if the threshold for clinical concerned be ‎reduced to 2µg/dL.‎
3. ‎Given the widespread presence of lead in the environment and its effect on ‎the children’s health, mothers should be educated about the harmful effects of ‎lead.‎
4. ‎Blood lead levels should be screened in 9 to 12 months old infants. ‎
5. The industrial areas have higher environmental pollution; and hence, should be ‎isolated from residential and educational areas.‎
6. Safe practices to reduce exposure to these pollutants in everyday life should ‎be implemented (e.g., labeling of products containing lead and not using the ‎metal household products, etc.).‎
7. Measuring and tracking blood lead levels of children at regular intervals to ‎reduce the harm caused by prolonged exposure to lead.‎
